# Counting using deep learning regression gives value to ecological surveys

**DOI:** 10.1038/s41598-021-02387-9

**Published:** 2021-12-01

**Authors:** Jeroen P. A. Hoekendijk, Benjamin Kellenberger, Geert Aarts, Sophie Brasseur, Suzanne S. H. Poiesz, Devis Tuia

**Affiliations:** 1 NIOZ Royal Netherlands Institute for Sea Research, 1790AB Den Burg, The Netherlands; 2 Wageningen University and Research, 6708PB Wageningen, The Netherlands; 3 Ecole Polytechnique Fédérale de Lausanne (EPFL), 1950 Sion, Switzerland; 4 Wageningen Marine Research, Wageningen University and Research, 1781AG Den Helder, The Netherlands; 5 Wageningen University and Research, Wildlife Ecology and Conservation Group, 6708 PB Wageningen, The Netherlands; 6 Groningen Institute of Evolutionary Life Sciences, University of Groningen, 9700 CC Groningen, The Netherlands

**Keywords:** Ecology, Ecology, Mathematics and computing

## Abstract

Many ecological studies rely on count data and involve manual counting of objects of interest, which is time-consuming and especially disadvantageous when time in the field or lab is limited. However, an increasing number of works uses digital imagery, which opens opportunities to automatise counting tasks. In this study, we use machine learning to automate counting objects of interest without the need to label individual objects. By leveraging already existing image-level annotations, this approach can also give value to historical data that were collected and annotated over longer time series (typical for many ecological studies), without the aim of deep learning applications. We demonstrate deep learning regression on two fundamentally different counting tasks: (i) daily growth rings from microscopic images of fish otolith (i.e., hearing stone) and (ii) hauled out seals from highly variable aerial imagery. In the otolith images, our deep learning-based regressor yields an *RMSE* of 3.40 day-rings and an $R^2$ of 0.92. Initial performance in the seal images is lower (*RMSE* of 23.46 seals and $R^2$ of 0.72), which can be attributed to a lack of images with a high number of seals in the initial training set, compared to the test set. We then show how to improve performance substantially (*RMSE* of 19.03 seals and $R^2$ of 0.77) by carefully selecting and relabelling just 100 additional training images based on initial model prediction discrepancy. The regression-based approach used here returns accurate counts ($R^2$ of 0.92 and 0.77 for the rings and seals, respectively), directly usable in ecological research.

## Introduction

Ecological studies aim to unravel the interactions between organisms and their environment at various spatial scales. In order to quantify these intricate relationships, many ecological studies rely on count data: for instance, during animal surveys, individuals are counted to estimate and monitor population size^1,2^ or to predict the spatial distribution of animals^3^. On smaller scales, counting physical traits is widely used in, for example, plant phenotyping, where the number of leaves of a plant is a key trait to describe development and growth^4,5^. The usage of count data in ecology is also common on microscopic scales, for example to estimate the age of fish by counting daily growth rings that are visible in otoliths (i.e., hearing stones)^6,7^.

Irrespective of the scale, counting objects of interest can be tedious and time-consuming, especially when objects occur in large numbers and/or densities (e.g., wildlife that clusters in colonies^8^), when they overlap (e.g., leaves of plants^4,5^), or when they are less well-defined and cryptic (e.g., otolith rings^6^). Historically, many of these traits were counted directly by eye. Later, objects of interest were photographed, which allowed for optimisation of the time in the field (or lab) and repeatability of the counts. Nowadays, many studies increasingly take advantage of digital photography, allowing for more efficient ways of archiving the data. Crucially, these archived images can now potentially be used for digital processing and automated counting.

To this end, recent ecological studies have shown promising potential of using computer vision to count objects of interest from digital imagery^9,10^: they employ Machine Learning (ML) models, which are trained on a set of manually annotated (labelled) images to learn to recognise patterns (e.g., colours and shapes), and eventually objects, in those training images. Once trained, these ML models can be used to automatically recognise similar patterns in new images and perform tasks like species classification, animal detection, and more^11^. Most successful ML models belong to the family of Deep Learning (DL)^12^, in particular Convolutional Neural Networks (CNNs)^13^.

Most ecological studies that use computer vision for counting apply CNNs designed for object detection^14–16^. These object detectors are trained on images in which every object of interest is annotated individually, most commonly by a bounding box drawn around the object, or a location point at its centre. Alternatively, objects of interest can be counted using detectors based on image segmentation^17^, which require even more extensive annotations, as every pixel in the image must be labelled. Annotating training images for object detection and image segmentation can therefore be labour-intensive, especially for images where object counts are high. Hence, this could potentially undermine the time (and cost) reduction advantage promised by ML models in the first place.

An alternative is to instead annotate training images with a single value that represents the number of objects in an image. These image-level annotations pose significantly reduced annotation time and can directly be used to train regression-based CNNs. Perhaps more importantly, image-level counts are an often used annotation format in ecological studies, for example in cases where objects are manually counted from digital imagery over longer time series. Furthermore, image-level annotations provide a viable solution for scenarios that are complicated to annotate otherwise, such as for overlapping objects^5^, complex and atypically shaped objects like concentric rings^18^, or continuous variables like an individual’s size or age^19^.

In this study we highlight the value of regression-based CNNs for ecological studies. We present a relatively lightweight DL model for counting objects in digital imagery and evaluate it on two fundamentally different real-world datasets, that were originally collected without the aim of training DL models. The first dataset consists of microscopic images of plaice (*Pleuronectes platessa*) otoliths (i.e., hearing stones) in which concentric rings are visible. These rings represent daily growth layers and are used to estimate the age of the fish to reconstruct egg and larval drift and calculate the contribution of various spawning grounds to different settling areas^6,7^. Plaice eggs and larvae are transported from their North Sea spawning grounds towards the coast of the North Sea and into the Wadden Sea (pelagic phase), where they settle (benthic phase). The transition of the pelagic phase to the benthic phase is visible in the otoliths. For this application, only the post-settlement benthic phase growth rings (visible directly after the pelagic phase centre) are counted. The already existing image-level annotations in this dataset are of high quality and are directly usable for DL applications. The second dataset consists of aerial images of grey seals (*Halichoerus grypus*) and harbour seals (*Phoca vitulina*) hauled out on land, which are collected from an aircraft using a hand-held camera during annual surveys monitoring population size and distribution^8^. These images are highly variable in light conditions, distance towards the seals, focal length and angle of view. For this second dataset, some of the existing image-level annotations were not directly usable for DL applications (see “Methods” section). Instead of recounting the seals and correcting the annotations for all images in this dataset, we propose a multi-step model building approach to handle scenarios where the quality of existing image-level annotations is insufficient to train a CNN. This approach can also be used to adapt the CNN to dataset variations that appear over time or with new acquisitions conditions.

These two real-world applications show that regression-based CNNs have the potential to greatly facilitate counting tasks in ecology. They allow researchers to reassign valuable resources and scale up their surveying effort, while potentially leveraging existing image-level annotations from archived datasets directly for the automation of counting.

## Results

For the results reported in this section, we used a pre-trained ResNet-18 CNN^20^ and modified it for the task of regression. After various experiments with other architectures and hyperparameters (Supplementary S1), we found that this relatively lightweight (i.e., shallow) ResNet-18, trained with a Huber loss function^21^ and with the largest possible batch size (limited by hardware, $n=84$ and $n=100$ images for the otolith and seal application, respectively) gave the best performance on the validation set, for both the seal and otolith ring counting application. Details on the CNN architecture selection and training are provided in the “Methods” section.

### Otolith daily growth rings from microscopic images

**Figure 1:**
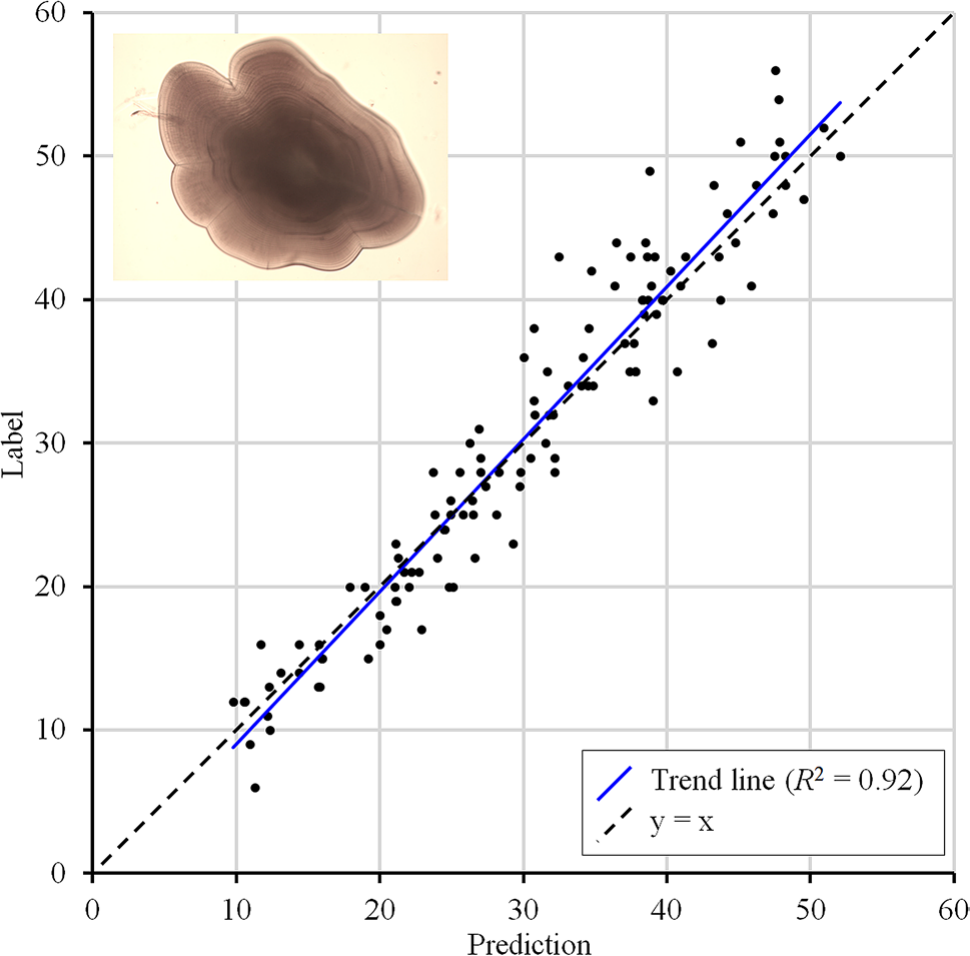
Numerical results on the otolith test set ($n=120$), where the labels (i.e., manual counts of post-settlement growth rings) are plotted against the predicted counts. The dotted line corresponds to the optimum $y=x$.

For the otolith growth ring counting application, the regression CNN was trained on 3465 microscopic images of otoliths. The results are provided in Fig. 1. Here, the predicted counts on the randomly selected test set ($n=120$) are plotted against the labels (i.e., the manual counts of the post-settlement growth rings). The CNN achieved an $R^2$ of 0.92, an *RMSE* of 3.40 day-rings and an *MAE* of 2.60 day-rings (Table 1), which corresponds to an average error of $9.9\%$.

### Hauled out seals from aerial images

**Figure 2:**
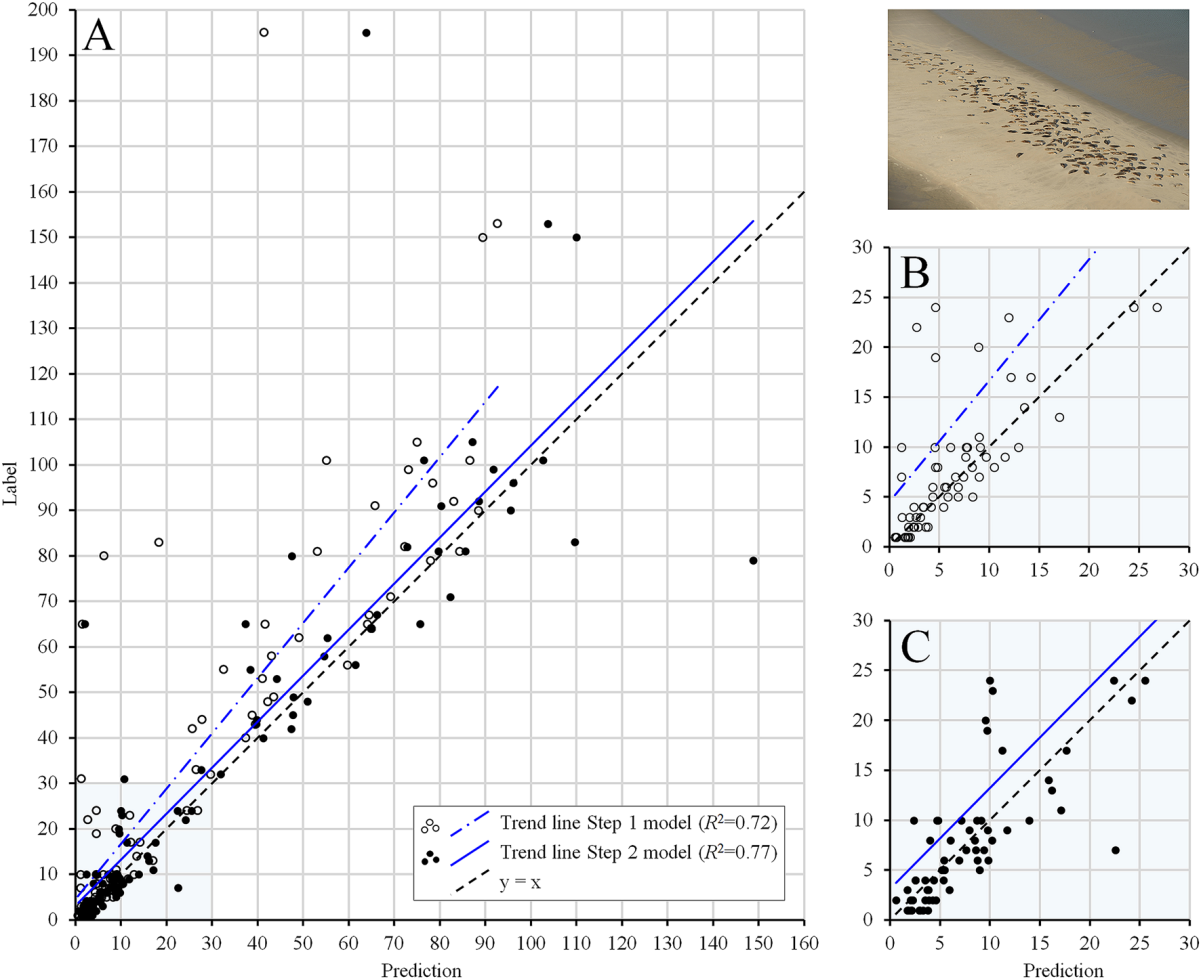
Numerical results on the seal test set ($n=100$), where the labels (i.e., the manual counts of hauled out seals) are plotted against the predicted counts. The black dotted lines resemble $y=x$. (**A**) The accuracy of the model trained on ‘seal subset 1’ (white dots) strongly improved after fine-tuning using training subset 2 (black dots). (**B**,**C**) Zoomed in (range 0-30) on predicted counts made by the Step 1 model (**B**) and the Step 2 model (**C**).

For the seal counting application, the existing image-level annotations were of insufficient quality (see “Methods” section) and manual recounting was required before training the CNN. Instead of recounting all the seals and correcting the annotations for all 11,087 aerial images in the main dataset, we applied a multi-step model building approach. First, two smaller subsets from the main dataset were selected, recounted and used for (i) a stratified random test set ($n=100$) and for (ii) training/validation (named ‘seal subset 1’, $n=787$) (see “Methods” section). Unlike the stratified random test set (which reflects the full distribution of available annotations from the main dataset), the images in ‘seal subset 1’ were selected (visually) for their high quality, which led to an under-representation of images with a high number of seals (which were generally of poorer quality). This first step greatly reduced the number of images that needed to be recounted and relabelled. Figure 2 (open dots, panels A and B) illustrates the predicted counts versus the real counts of the resulting model. This Step 1 model achieved an $R^2$ of 0.72, an *RMSE* of 23.46 seals and an *MAE* of 10.47 seals on the seal test set (Table 1). The next step allowed us to focus on images where the CNN was most incorrect. Here, the Step 1 model was used to predict counts on the 10,200 remaining images from the main dataset (that still include noisy labels). To train the model further, the images from the main dataset in which the number of seals was most overestimated ($n=50$) and most underestimated ($n=50$) with respect to the original (noisy) labels were selected (‘seal subset 2’), manually recounted and relabelled and used to supplement ‘seal subset 1’. By further tuning the model using this extended training/validation set, the performance on the test set improved (Fig. 2, solid dots, panel A and C), with the model achieving an $R^2$ of 0.77, an *RMSE* of 19.03 seals and an *MAE* of 8.14 seals (Table 1). This can be attributed mostly to improved predictions for images with a higher number of seals. Experiments with a random sampling on the whole distribution of labels (i.e., 787 images randomly selected from ‘seal subset 1’ and ‘seal subset 2’ combined, including images with a high number of seals) did not lead to better performance of the Step 1 model (see Supplementary S3). Thus, the two-step strategy allowed us to significantly improve the model performance on the seals with only 100 images to be re-annotated, thereby reducing labelling efforts to a minimum.

In the test set, a total of 3300 seals were annotated. With our multi-step approach, the predicted total number of seals on the test set increased from 2372 (71.9% of the total) to 2986 (90.5%) for the Step 1 and Step 2 model, respectively.

**Table 1:** Numerical performance of the proposed method on the randomly selected test sets for both applications. The performance of the seal counting application increased after fine-tuning the Step 1 model using ‘seal subset 2’ (Step 2 model).

	Otolith	Seals	
	Rings	Step 1 model	Step 2 model
$R^2$	0.92	0.72	0.77
*RMSE*	3.40	23.46	19.03
*MAE*	2.60	10.47	8.14

### Visualising counts

**Figure 3:**
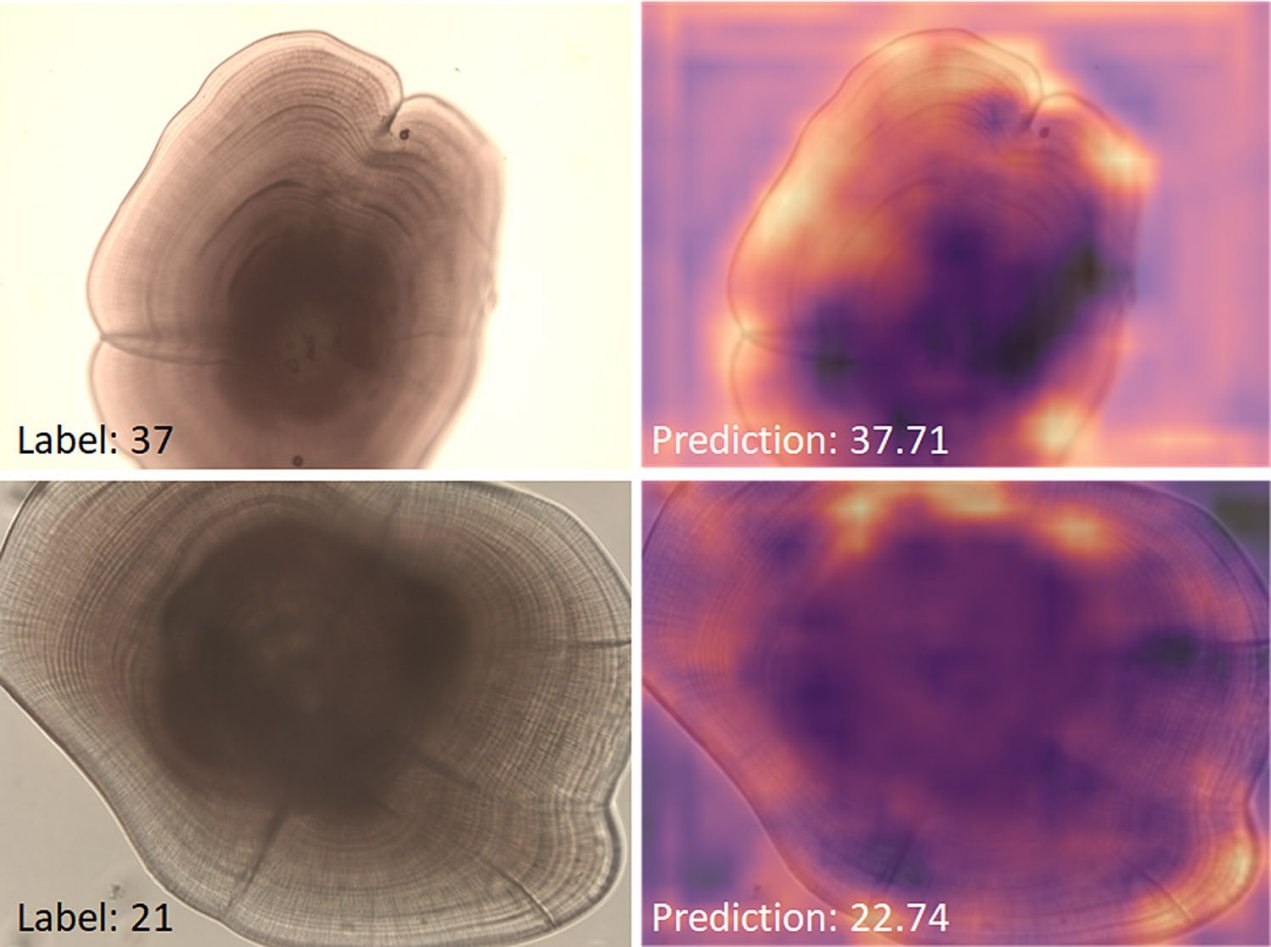
Examples of images (left) and CAMs (right), with good performance from the ring test set.

Class activation maps (CAM)^22,23^ of images from the test sets were used to further examine model performance. These heatmaps represent the regions of the original image that contributed the most to the final prediction of the CNN. The heatmaps of the otolith images (Fig. 3) were less informative than those of the the seal images. However, they illustrate that areas with more contrasting post-settlement rings were highlighted, while the accessory growth centre (containing pre-settlement growth rings that are not targeted by this application) did not seem to contribute to the prediction (i.e., it remained darker). This underlines that the model is indeed focusing on the task of counting post-settlement growth rings.

**Figure 4:**
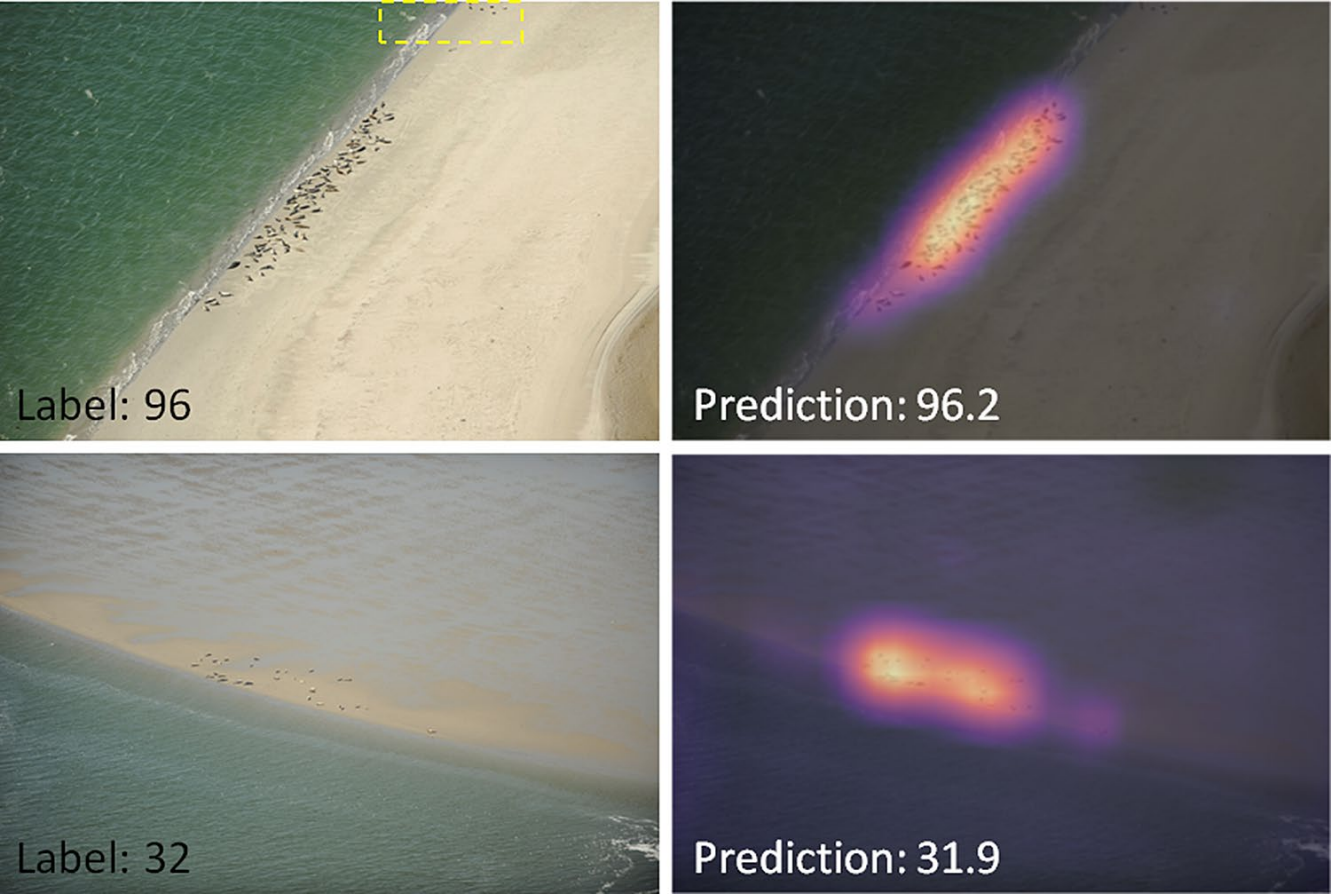
Examples of images (left) and CAMs (right), with good performance from the seal test set. Notice that in the top example some birds are visible (yellow dotted line), which are not counted by the model, which has specialised on seals.

For most seal images, the heatmaps show that the regions containing seals contributed the most to the final prediction. Unlike the cryptic concentric otolith rings, seals are clearly picked up by the model, according to the heatmaps (Fig. 4).

## Discussion

The regression-based CNN presented here performed well when trained on the two fundamentally different datasets. This was achieved without making any modifications to the architecture of the CNN between the two cases, except for training hyperparameters like the learning rate and number of epochs (see “Methods” section). By automating the counting tasks, the processing time of newly acquired images is dramatically reduced: processing 100 images using our trained CNN takes less than a minute, while manual processing the same amount of images is estimated to require at least one hour for the seals and three hours for the otoliths.

The accuracies reported here are directly usable in ecological research. For harbour seals, a correction factor of 0.68 is routinely used to extrapolate the survey counts to a population size estimate^24^. The 95% confidence interval of this correction factor is [0.47, 0.85]. In other words, the uncertainty in the population size estimate is minus 21% or plus 17%, which is substantially larger than the 9.5% underestimate in the total predicted counts of our Step 2 model. For the ring counting application, a coefficient of variation between multiple human experts was not available for daily growth rings of plaice. However, these are reported for yearly growth rings of Greenland halibut as 12%^25^ and 16.3%^26^, which is higher than the reported 9.9% average error obtained by our deep counting regression approach.

The two datasets feature different challenges regarding both the quality of the existing annotations and the task complexity. In the case of the otoliths, the existing annotations were of good quality and could be used directly to train the model. These image-level annotations provide a solution to label the complex concentric growth rings, which would be extremely difficult to annotate using other approaches, such as bounding boxes. A DL regression-based approach was applied in previous research to count otolith growth rings^18^, which achieved a higher accuracy on their test-set (*MSE* of 2.99). However, the tasks considered in that study were radically different from ours: in their paper, Moen and colleagues^18^ considered year rings, which are less cryptic than the post settlement day rings considered in this paper. Furthermore, our model was trained with fewer images ($n=3585$ instead of $n=8875$), to make predictions on a wider range of counts (1 to 63 day-rings instead of 1 to 26 year-rings). Finally, we evaluated the performance using a stratified random test set, which covers the ensemble of the distribution of possible values, while Moen and colleagues used a non-stratified test set, therefore reducing the number of occurrences of rare out-of-distribution cases in the test set.

In the case of the seals, the counting task was complex due to the high variability of the images (e.g., lighting conditions, distance from the seals and angle of view). Additionally, some of the existing count labels were not directly usable for training a CNN (see “Methods” section). However, this provided an opportunity to demonstrate the use of an iterative approach, in which the required re-annotation efforts could be minimised and focused on images where the model performed poorly. The CNN was first trained using only a subset containing recounted high quality images (‘seal subset 1’). As is common among DL applications, the resulting Step 1 model performed relatively poorly when it needed to make predictions that fell outside the range of the training images. This was the case for images in which a high number of seals were visible and/or when the seals appeared smaller (i.e., were photographed from a larger distance or a smaller focal length was used). The poor performance on these type of images could be attributed to ‘seal subset 1’ containing only images with clearly visible seals, ranging from zero to 99 individuals (see “Methods” section). By using the Step 1 model predictions to guide the selection of images that need to be reviewed, a relatively small number of images (‘seal subset 2’) was selected from the remaining images in the main database, to supplement ‘seal subset 1’. This multi-step approach allows to focus on images with a large potential for improvement for the Step 2 model: many of the images in ‘seal subset 2’ contained a high number of seals and/or seals that appeared smaller. This approach can therefore also be used to cope with dataset variations that appear over time or with new acquisitions conditions. The high variability in the seal dataset (i.e., distance towards seals, angle of view and zoom level) suggests that a regression-based approach based on this data can also provide solutions for scenarios where the objects of interest move through a three-dimensional space (e.g., flocks of birds, schools of fish), provided that the model is trained with a wide variety of input data covering the expected variations.

**Figure 5:**
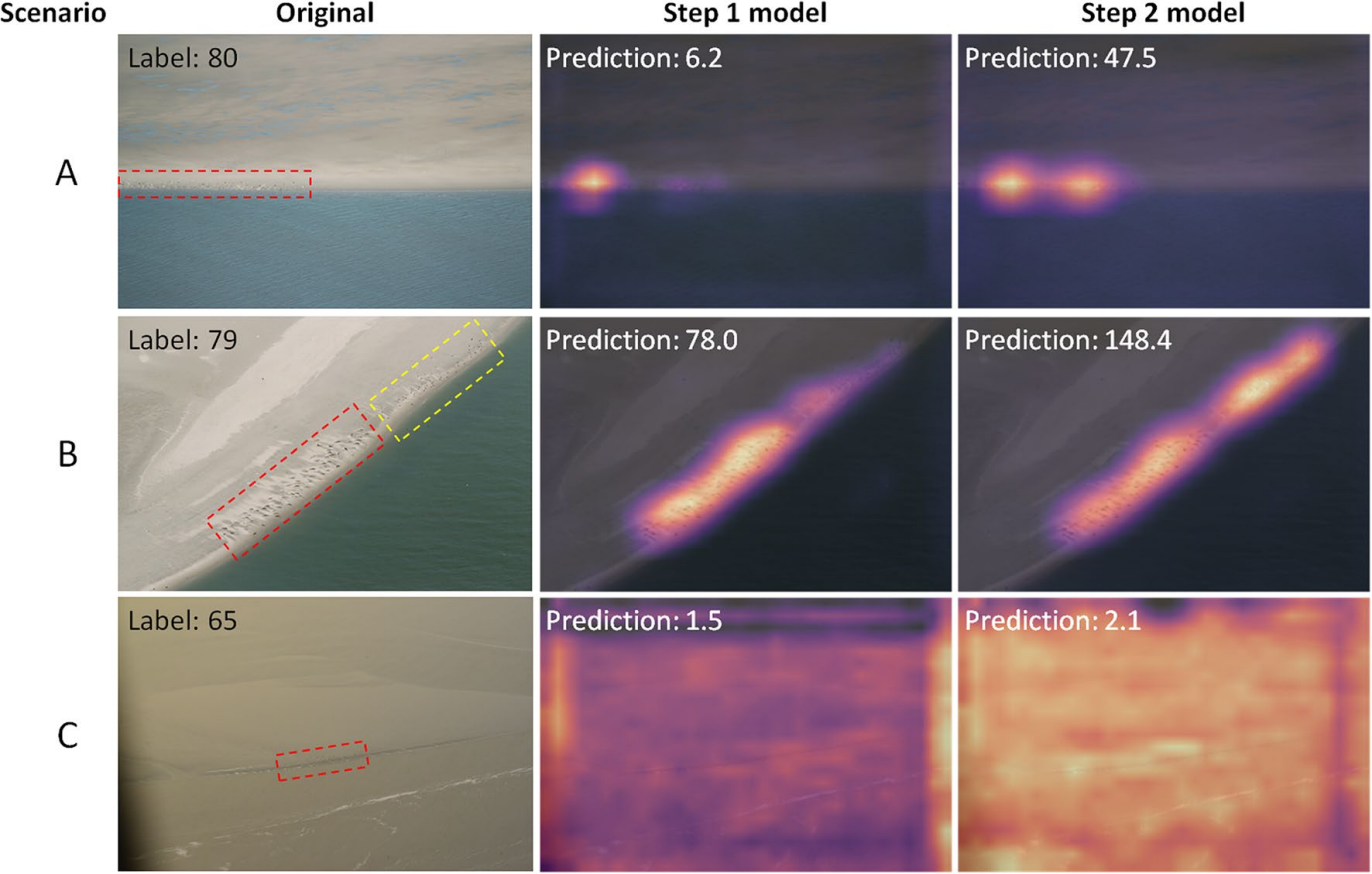
Examples of CAMs for cases with unsatisfactory performance. The first column shows the unedited aerial images, where the red dotted line marks the area where the seals are visible. The second and third columns show the heatmaps when predictions are made using the Step 1 and Step 2 model, respectively. For case (**A**) (small seals) the performance increased, but is still unsatisfactory, as seals remain only partially detected. For case (**B**) the performance decreased as birds (yellow dotted line) start to contribute to the predictions, while for case (**C**) (blurry and extremely small seals) the performance was poor for both models.

In contrast with an object detection approach, it is not possible to evaluate the predicted location of single objects in our regression-based approach, as the predictions are given as image-level counts. However, by using CAMs as presented here, model decisions can be visualised and used to evaluate the model performance in more detail. In case of the seals, these heatmaps were used to further compare the performance of the Step 1 and Step 2 model on the test set. The Step 2 model generally performed better, especially for images where seals appeared smaller (e.g., Fig. 5, case A). For some images however, the model predictions deteriorated. This was for instance the case for an image with birds presents adjacent to the seals, which contributed to the predicted counts for the Step 2 model (Fig. 5, case B). For some images that were particularly difficult (e.g., due to blur or extremely small seals), the Step 2 model remained unable to count seals adequately (Fig. 5, case C).

For future applications, automated counts based on the regression approach presented here could potentially be further improved by changing the survey design to have lower variability in the images. In the case of the seals for instance, this could be obtained by photographing the seals from a more constant distance with a single focal length, although in practice this might be challenging. For existing data sets, the model could also deliberately be exposed to more appearance variability. This could for instance be done by resorting to un- or semi-supervised domain adaptation routines^27^. This requires no or only a few extra annotated images but result in more robustness of the model to the appearance variations inherent in the data. Alternatively, in cases such as the seals, where many images remain unused due to noisy labels, the iterative approach presented in this study could be repeated, which is expected to further improve the models performance.

In many computer vision disciplines, regression-based CNNs similar to the one employed here are commonly used for counting tasks, especially when the objects of interest occur in high densities and high numbers, such as human crowds^28,29^ or buildings^30^. They have also been used in some ecological applications, particularly when the objects of interest are hard to annotate using bounding boxes, for instance in the case of overlapping plant leaves^5,31,32^. Wildlife counting is a domain that is typically addressed with spatially explicit object detection approaches^14,15^. Few other works have addressed this task using regression-based CNNs^33,34^, but they either had no explicit focus on wildlife detection^34^ or used it to approximate spatial locations^33^. Nonetheless, proceeding with a regression approach permits to process surveys where only global counts are provided, rather than precise annotations of individuals that would be required by object detection approaches. But even in the presence of individual annotations, the regression approach remains competitive in terms of final counts: when compared to a traditional deep object detection approach (Faster R-CNN^35^) on a manually annotated subset of the seals dataset, our regression approach remained more accurate (details in Supplementary S2). Furthermore, it took approximately one hour to obtain the image-level annotations required to train the regression-based CNN, while it took over 8 hours to create the individual bounding boxes required to train the Faster R-CNN model.

Our study illustrates how a relatively lightweight regression CNN can be used to automatically count objects of interest from digital imagery in fundamentally different kinds of ecological applications. We have shown that it is well-suited to count wildlife (especially when individuals occur in high densities) and to count cryptic objects that are extremely difficult to annotate individually. Previous ecological studies have shown that by automating detection tasks, time and resources can be reassigned, allowing for an increase in sampling effort^14^. By using annotations at the image-level, labelling efforts and costs can be reduced. Finally, a unique advantage of using a regression-based approach is that it has the potential to leverage already existing labels, collected without the aim of DL applications, thereby reducing labelling efforts and costs to zero.

## Methods

### Datasets

In this study, datasets from two fundamentally different real-world ecological use cases were employed. The objects of interest in these images were manually counted in previous studies^2,8,36,37^, without the aim of DL applications.

#### Microscopic images of otolith rings

**Figure 6:**
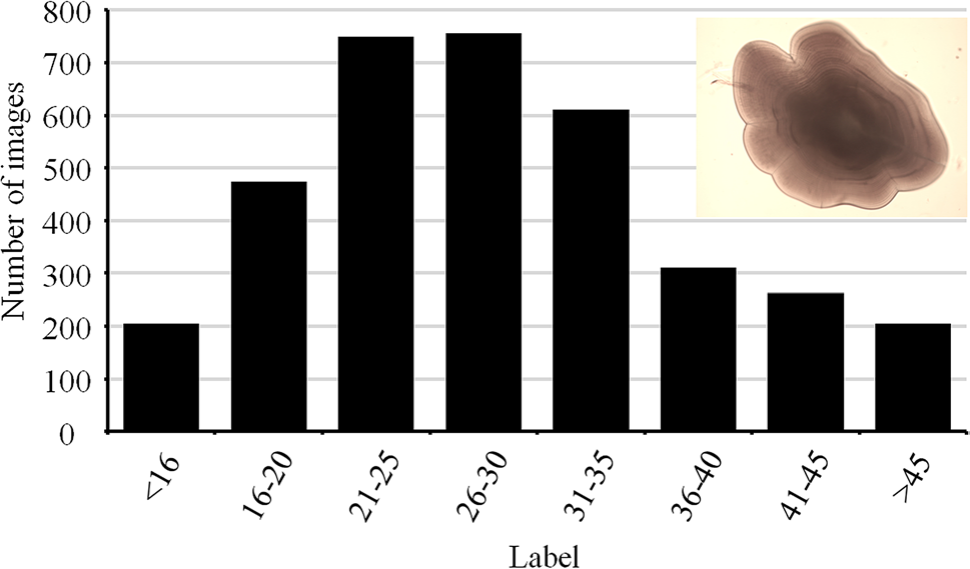
Distribution of the labels (i.e., number of post-settlement rings) of all images in the otolith dataset ($n=3585$).

The first dataset consists of 3585 microscopic images of otoliths (i.e., hearing stones) of plaice (*Pleuronectes platessa*). Newly settled juvenile plaice of various length classes were collected at stations along the North Sea and Wadden Sea coast during 23 sampling campaigns conducted over 6 years. Each individual fish was measured, the sagittal otoliths were removed and microscopic images of two zoom levels ($10\times 20$ and $10\times 10$, depending on fish length) were made. Post-settlement daily growth rings outside the accessory growth centre were then counted by eye^6,7^. In this dataset, images of otoliths with less than 16 and more than 45 rings were scarce (Fig. 6). Therefore, a stratified random design was used to select 120 images to evaluate the model performance over the full range of ring counts: all 3585 images were grouped in eight bins according to their label (Fig. 6) and from each bin 15 images were randomly selected for the test set. Out of the remaining 3465 images, 80% of the images were randomly selected for training and 20% were used as a validation set, which is used to estimate the model performance and optimise hyperparameters during training.

#### Aerial images of seals

**Figure 7:**
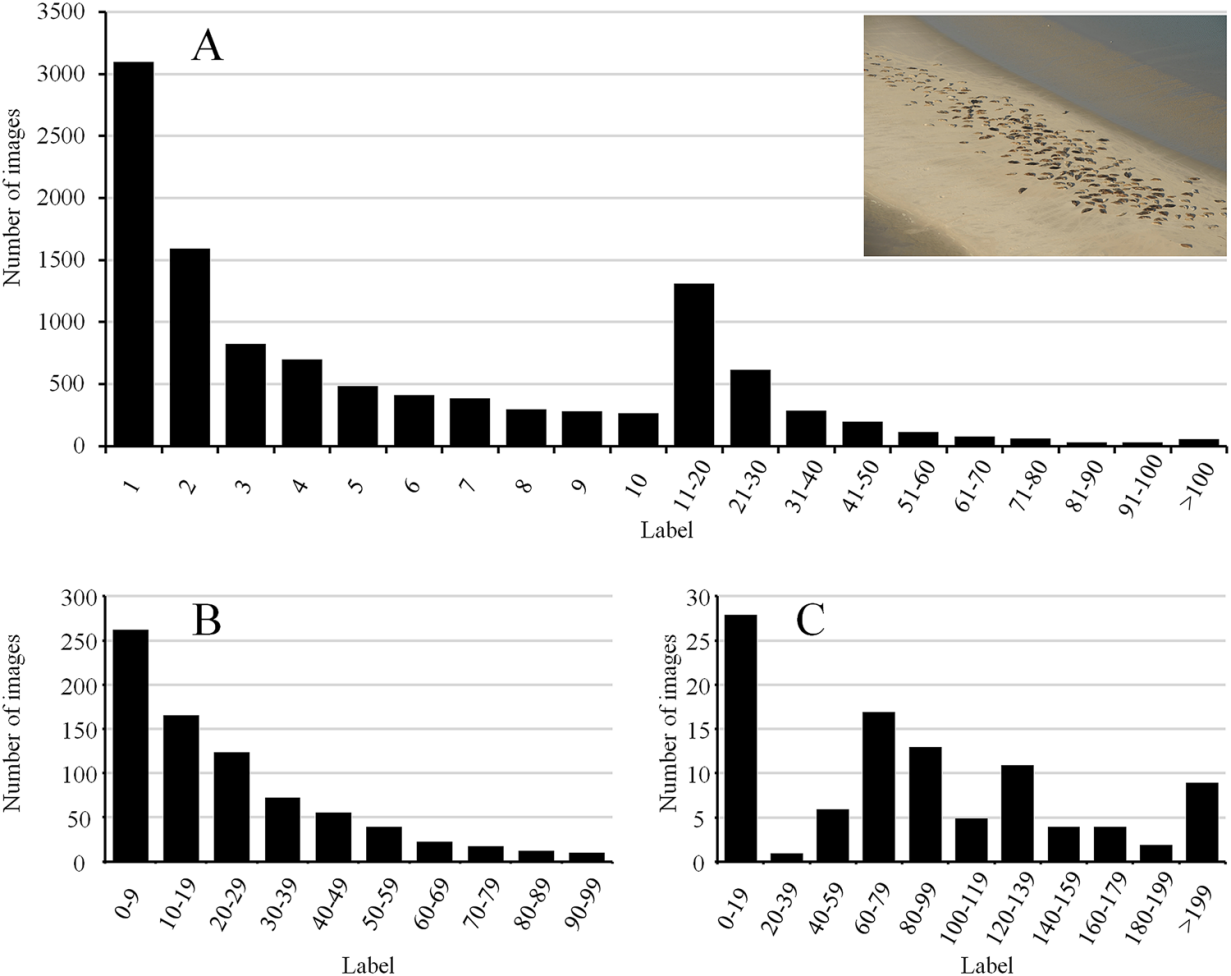
Distribution of the labels (i.e., number of seals) in (**A**) the seal main dataset ($n=11{,}087$), (**B**) ‘seal subset 1’ ($n=787$) and (**C**) ‘seal subset 2’ ($n=100$).

The second dataset consists of 11,087 aerial images (named ‘main dataset’ from now onwards) of hauled out grey seals (*Halichoerus grypus*) and harbour seals (*Phoca vitulina*), collected between 2005 and 2019 in the Dutch part of the Wadden Sea^2,36^. Surveys for both species were performed multiple times each year: approximately three times during pupping season and twice during the moult^8^. During these periods, seals haul out on land in larger numbers. Images were taken manually through the airplane window whenever seals were sighted, while flying at a fixed height of approximately 150m, using different focal lengths (80-400mm). Due to variations in survey conditions (e.g., weather, lighting) and image composition (e.g., angle of view, distance towards seals), this main dataset is highly variable. Noisy labels further complicated the use of this dataset: seals present in multiple (partially) overlapping images were counted only once, and were therefore not included in the count label of each image. Recounting the seals on all images in this dataset to deal with these noisy labels would be a tedious task, compromising one of the main aims of this study of reducing annotation efforts. Instead, only a selection of the main dataset was recounted and used for training and testing. First, 100 images were randomly selected (and recounted) for the test set. In the main dataset, images with a high number of seals were scarce, while images with a low number of seals were abundant (Fig. 7, panel A). Therefore, as with the otoliths, all 11,087 images were grouped into 20 bins according to their label (Fig. 7, panel A), after which five images were randomly selected from each bin for the test set. Second, images of sufficient quality and containing easily identifiable were selected from the main dataset (and recounted) for training and validation, until 787 images were retained (named ‘seal subset 1’). In order to create images with zero seals (i.e., just containing the background) and to remove seals that are only partly photographed along the image borders, some of these images were cropped. The dimensions of those cropped images were preserved and, if required, the image-level annotation was modified accordingly. The resulting ‘seal subset 1’ only contains images with zero to 99 seals (Fig. 7, panel B). These 787 images were then randomly split in a training (80%) and validation set (20%). In order to still take advantage of the remaining 10,200 images from the main dataset, a two-step label refinement was performed (see the section “Dealing with noisy labels: two-step label refinement” below).

### Convolutional neural networks

CNNs are a particular type of artificial neural network. Similar to a biological neural network, where many neurons are connected by synapses, these models consist of a series of connected artificial neurons (i.e., nodes), grouped into layers that are applied one by one. In a CNN, each layer receives an input and produces an output by performing a convolution between the neurons (now organised into a rectangular filter) and each spatial input location and its surroundings. This convolution operator computes a dot product at each location in the input (image or previous layer’s output), encoding the correlation between the local input values and the learnable filter weights (i.e., neurons). After this convolution, an activation function is applied so that the final output of the network can represent more than just a linear combination of the inputs. Each layer performs calculations on the inputs it receives from the previous layer, before sending it to the next layer. Regular layers that ingest all previous outputs rather than a local neighbourhood are sometimes also employed at the end; these are called “fully-connected” layers. The number of layers determines the depth of the network. More layers introduce a larger number of free (learnable) parameters, as does a higher number of convolutional filters per layer or larger filter sizes. A final layer usually projects the intermediate, high-dimensional outputs into a vector of size *C* (the number of categories) in the case of classification, into a single number in the case of regression (ours), or into a custom number of outputs representing arbitrarily complex parameters, such as the class label and coordinates of a bounding box in the case of object detection. During training, the model is fed with many labelled examples to learn the task at hand: the parameters of the neurons are updated to minimise a loss (provided by an error function measuring the discrepancy between predictions and labels; in our case this is the Huber loss as described below). To do so, the gradient and its derivative with respect to each neuron in the last layer is computed; modifying neurons by following their gradients downwards allows reducing the loss (and thereby improving model prediction) for the current image accordingly. Since the series of layers in a CNN can be seen as a set of nested, differentiable functions, the chain rule can be applied to also compute gradients for the intermediate, hidden layers and modify neurons therein backwards until the first layer. This process is known as backpropagation^38^. With the recent increase of computational power and labelled dataset sizes, these models are now of increasing complexity (i.e., they have higher numbers of learnable parameters in the convolutional filters and layers).

CNNs come in many layer configurations, or architectures. One of the most widely used CNN architecture is the ResNet^20^, which introduced the concept of residual blocks: in ResNets, the input to a residual block (i.e., a group of convolutional layers with nonlinear activations) is added to its output in an element-wise manner. This allows the block to focus on learning residual patterns on top of its inputs. Also, it enables learning signals to by-pass entire blocks, which stabilises training by avoiding the problem of vanishing gradients^39^. As a consequence, ResNets were the first models that could be trained even with many layers in series and provided a significant increase in accuracy.

### Model selection and training

For the otolith dataset, we employed ResNet^20^ architectures of various depths (i.e., ResNet18, ResNet34, ResNet50, ResNet101 and ResNet152, where the number corresponds to the number of hidden layers in the model, see Supplementary S1). These ResNet models were pretrained on ImageNet^40^, which is a large benchmark dataset containing millions of natural images annotated with thousands of categories. Pre-training on ImageNet is a commonly employed methodology to train a CNN efficiently, as it will already have learned how to recognise common recurring features, such as edges and basic geometrical patterns, which would have to be learned from zero otherwise. Therefore, pre-training reduces the required amount of training data significantly.

**Figure 8:**
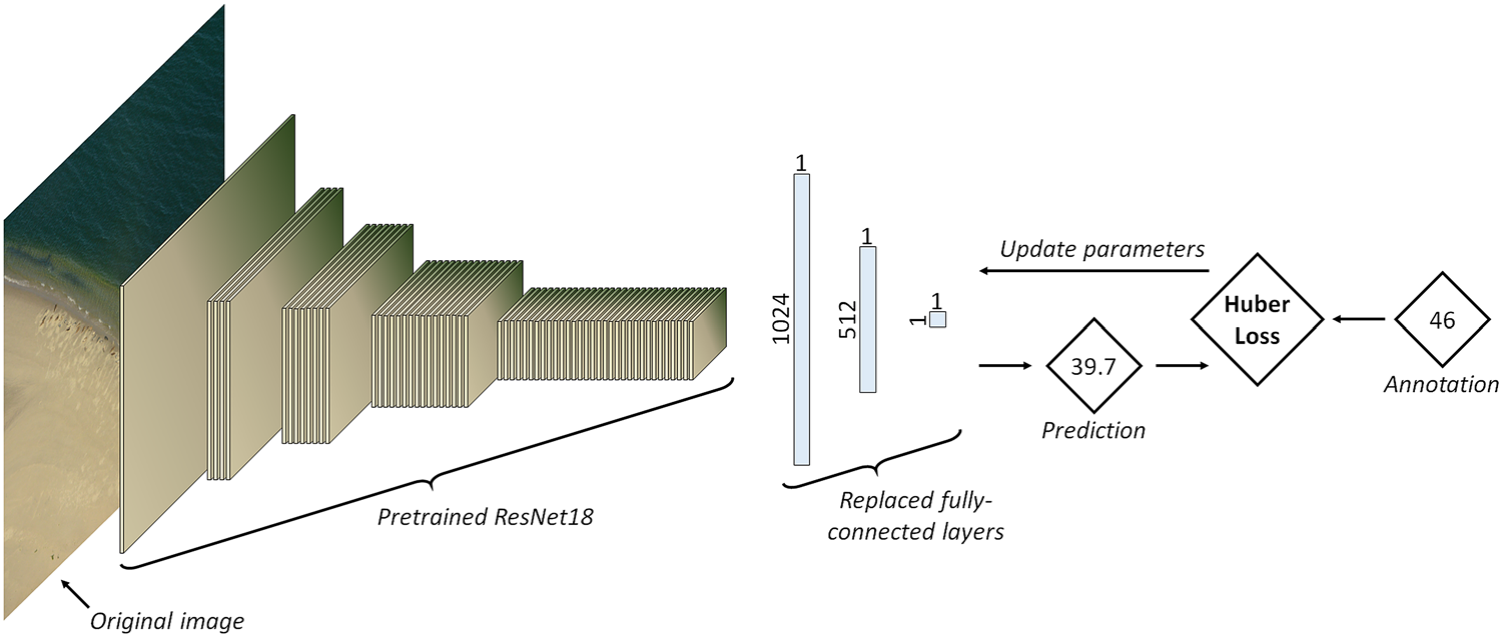
Schematic representation of the CNN used in this study. The classification output layer of the pretrained ResNet18 is replaced by two fully-connected layers. The model is trained with a Huber loss.

We modified the ResNet architecture to perform a regression task. To do so, we replaced the classification output layer with two fully-connected layers that map to 512 neurons after the first layer and to a single continuous variable after the second layer^23^ (Fig. 8). Since the final task to be performed is regression, the loss function is a loss function that is tailored for regression. In our experiments we tested both a Mean Squared Error and a Smooth L1 (i.e., Huber) loss^21^ (see Supplementary S1). The Huber loss is more robust against outliers and is defined as follows:

1 $$\begin{aligned} {\mathscr {L}}(y,{\hat{y}})=\frac{1}{n}\sum _i^{n} z_i \end{aligned}$$

where $z_i$ is given by

2 $$\begin{aligned} z_i= {\left\{ \begin{array}{ll} 0.5\times (y_i-{\hat{y}}_i)^2, &{}\quad \text {if } |y_i-{\hat{y}}_i|<1\\ |y_i-{\hat{y}}_i|-0.5, &{}\quad \text {otherwise} \end{array}\right. } \end{aligned}$$

where ${\hat{y}}$ is the value predicted by the model, *y* is the true (ground truth) value (i.e., the label) and *n* is the batch size. Intuitively, the Huber loss assigns a strong (squared) penalty for predictions that are close to the target value, but not perfect (i.e., loss value $<1$) and a smaller (linear) penalty for predictions far off, which increases tolerance towards potential outliers both in prediction and target.

Computations were performed on a Linux server with four Nvidia GeForce GTX 1080 Ti graphics cards. The CNNs were trained using the FastAI library^23^ (version 2.0.13) in PyTorch^41^ (version 1.6.0). FastAI’s default settings were used for image normalisation, dropout^42^, weight decay and momentum^23^, and a batch size of 84 images was used for the otolith dataset. Whenever an image was used in a model iteration during training, a series of transformations was applied randomly to it for data augmentation (including resizing to $1040\times 770$ pixels, random horizontal flips, lighting, warping, zooming and zero-padding). When using image-level annotations, only limited degrees of zooming can be used, otherwise objects of interest might be cut out of the image, making the image-level annotations incorrect. For the same reason, images were squeezed instead of cropped whenever necessary to account for different image dimensions. Various Learning Rates (LR) and Batch Sizes (BS) were evaluated (see Supplementary S1). A LR finder^43^ was used to determine the initial LR values, and FastAIs default settings for discriminative LR were applied^23^. In discriminative LR, a lower LR is used to train the early layers of the model, while the later layers are trained using a higher LR. For this purpose, our model was divided into three sections (the pretrained part of the network is split into two sections, while the third section comprised the added fully-connected layers), that each had a different LR (specified below) during training. Additionally, we applied ‘1cycle training’^23,44^. Here, training is divided into two phases, one where the LR grows towards a maximum, followed by a phase where the LR is reduced to the original value again. Firstly, only the two fully-connected layers added for regression (i.e., the third section) were trained for 25 epochs (of which the best performing 24th epoch was saved) with an LR of $5e-2$, while the rest of the network remained frozen. After this, the entire network was unfrozen and all layers were further tuned using a discriminative LR ranging from $9e-7$ to $9e-5$, for another 50 epochs, of which the best performing epoch was saved (50th epoch). The same model architecture, training approach and hyperparameters were used for the seal images, with the following exceptions. The batch size was 100 and images were resized to to $1064\times 708$ pixels. First, only the added layers were trained (analogue to the rings), with an LR of $3e-2$, for 50 epochs (of which the best performing 45th epoch was saved). After this, the entire network was unfrozen and further tuned for 50 epochs (of which the best performing epoch, the 49th, was saved), using a discriminative LR ranging from $3e-4$ to $3e-2$.

For both the otolith and seal cases, the trained models were evaluated on their respective test sets (described above). These test sets represent unseen data that is not used during the training and validation of the model. $R^2$, *RMSE* and *MAE* were used as performance metrics, and predicted counts were plotted against the labels. Additionally, Class Activation Maps (CAM) were made to aid with interpreting the models predictions^22,23^.

### Dealing with noisy labels: two-step label refinement

In order to take advantage of the additionally available noisy data during training, a two-step approach was employed that avoids the need to recount tens of thousands of seals. By using the Step 1 model (trained using ‘seal subset 1’) predictions, an additional 100 images were selected (and recounted) from the remaining main dataset (see “Results” section). For 35 images, the seals were not clearly identifiable by eye (i.e., they appeared too small) and the image was discarded and replaced by the next most poorly predicted image. These resulting 100 images (named ‘seal subset 2’, Fig. 7, panel C) were expected to include cases with noisy labels, but also cases that were challenging for the model to predict (e.g., images with a high number of seals). After this, the entire model (i.e., all layers) was retrained using ‘seal subset 1’ supplemented with ‘seal subset 2’, randomly split in a training (80%) and validation set (20%), for an additional 50 epochs using the same hyperparameters as before, except for the LR. Various LR were evaluated and a discriminative LR ranging from $1e-5$ to $1e-3$ gave the best performance on the validation set, in the 48th epoch.

## Supplementary Information

Supplementary Information 1.

Supplementary Information 2.

Supplementary Information 3.

## Data Availability

The data used in this study are open-source and publicly available. Code and data associated with this study can be obtained at 10.25850/nioz/7b.b0c.
