# Supplementary material for: Counting using deep learning regression gives value to ecological surveys
Source: Sci Rep. 2021 Dec 1;11:23209. doi: 10.1038/s41598-021-02387-9 (PMC8636638; doi:10.1038/s41598-021-02387-9)
Supplement: Supplementary file 1 — Supplementary Information 1. [file 41598_2021_2387_MOESM1_ESM.pdf]

Counting using deep learning regression gives value to ecological surveys.

Jeroen PA Hoekendijk, Benjamin Kellenberger, Geert Aarts, Sophie Brasseur, Suzanne SH Poiesz, and Devis Tuia.

Supplementary materials S1

ResNet	Loss	BS	RMSE
18	Huber	84	2.47
18	Huber	16	3.69
34	Huber	64	2.55
34	MSE	64	2.58
34	Huber	32	2.43
34	MSE	32	2.54
34	Huber	16	3.66
50	Huber	16	3.34
101	Huber	16	3.33
152	Huber	12	4.03

Supplementary Table S1.1. Numerical results on the validation set of the otolith ring counting application, using various ResNet architectures, loss functions (Huber vs. MSE) and batchsizes (BS).

ResNet	Loss	BS	RMSE
18	Huber	100	4.48
18	Huber	64	4.38
18	Huber	16	5.21
34	Huber	64	5.50
34	MSE	64	5.45
34	Huber	32	5.28
34	MSE	32	7.62
34	Huber	16	10.01
50	Huber	16	7.06
101	Huber	16	8.84
152	Huber	12	9.90

Supplementary Table S1.2. Numerical results on the validation set of the seal counting application, using various ResNet architectures, loss functions (Huber vs. MSE) and batchsizes (BS).
