# Supplementary material for: Counting using deep learning regression gives value to ecological surveys
Source: Sci Rep. 2021 Dec 1;11:23209. doi: 10.1038/s41598-021-02387-9 (PMC8636638; doi:10.1038/s41598-021-02387-9)
Supplement: Supplementary file 2 — Supplementary Information 2. [file 41598_2021_2387_MOESM2_ESM.pdf]

# Counting using deep learning regression gives value to ecological surveys.

Jeroen PA Hoekendijk, Benjamin Kellenberger, Geert Aarts, Sophie Brasseur, Suzanne SH Poiesz, and Devis Tuia.

## Supplementary materials S2

Wildlife counting is a domain that is typically dominated by object detection approaches. In this Supplementary materials S2, we compare the predicted counts on our 'seal test set' of an object detector (Faster R-CNN) with a regressor (as described in the main text for the 'Step 1 model'), both trained on a small subset of the available data.

Training an object detector requires individual seals to be annotated. Instead of annotating the entire seal dataset (987 images, containing 31,419 seals), we created a sub-set of 100 training images, randomly selected from 'seal subset 1' and 'seal subset 2' combined. This new sub-set contained 3,021 individual seals, that were then annotated individually using bounding boxes. Using these 100 images, we first trained a Faster R-CNN (Ren *et al.*, 2015) on the bounding boxes directly. In detail, we split the images into non-overlapping patches of size 224x224 and trained on 16 patches at a time. We employed a ResNet-50 FPN as backbone and trained the model for 300,000 iterations with stochastic gradient descent and an initial learning rate of 0.01, divided by 10 after 100,000 and 200,000 iterations, respectively. During testing, we also predicted on patches of the same size and recorded the number of seals detected across all patches per image. Additionally, we trained a regressor (as described in main text for the 'Step 1 model') on the same 100 images and compared the predicted counts on the 'Seal test set'. The Faster R-CNN achieved an $R^2$ of 0.38 and an RMSE of 46.5. The poorest predictions were on images with a high number of (small) seals and images that contained many birds. The regressor performed significantly better with an RMSE of 32.2, although the $R^2$ was equivalent (0.36). The results of this small-scale experiment are indicative that -for the seal application- a regression-based approach as the one presented here is a valid alternative for a detection/classification network. Furthermore, the annotation process by drawing bounding boxes took slightly over 8 hours, while obtaining image-level counts by manual counting took slightly over one hour.

| | Faster R-CNN | Regressor |
|---|---|---|
| **$R^2$** | 0.38 | 0.36 |
| **RMSE** | 46.5 | 32.2 |

**Supplementary Table S2.1**. Numerical results on the test set of the seal counting application, using an object detector (faster R-CNN) and a regressor (as described in the main text for the 'Step 1 model')

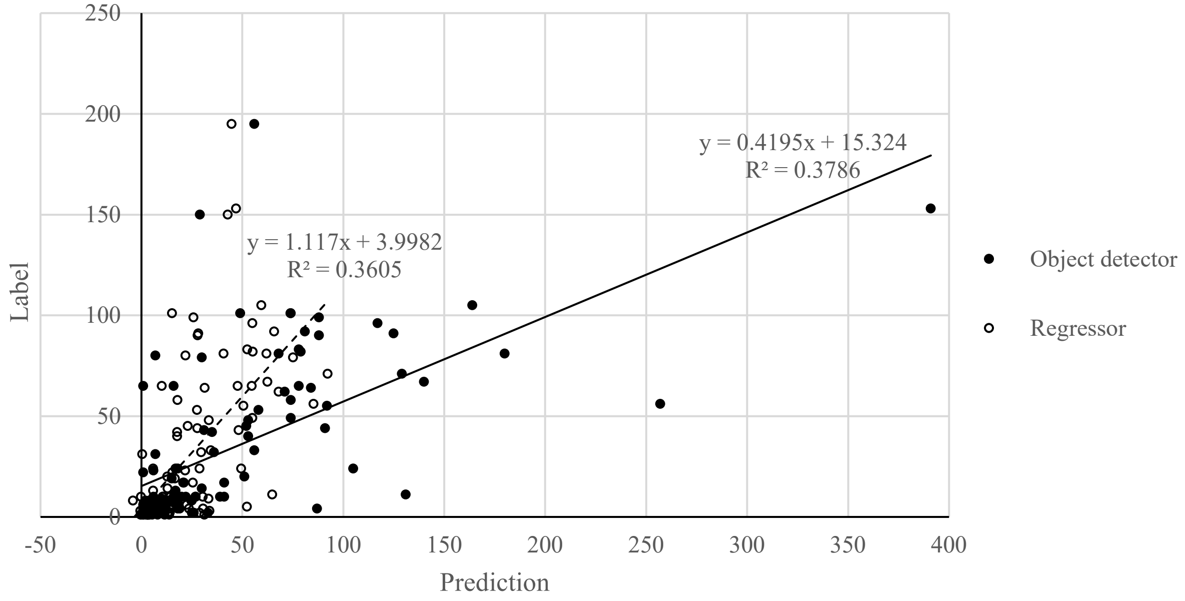


**Supplementary Figure S2.1**. Numerical results on the test set of the seal counting application, where the labels (i.e., the manual counts of hauled out seals) are plotted against the predicted counts, using an object detector (Faster R-CNN, black dots) and a regressor (as described in the main text for the ‘Step 1 model’, white dots). Both are trained using a small subset (n = 100) of randomly selected images from ‘seal subset 1’ and ‘seal subset 2’ combined.
