# Supplementary material for: Counting using deep learning regression gives value to ecological surveys
Source: Sci Rep. 2021 Dec 1;11:23209. doi: 10.1038/s41598-021-02387-9 (PMC8636638; doi:10.1038/s41598-021-02387-9)
Supplement: Supplementary file 3 — Supplementary Information 3. [file 41598_2021_2387_MOESM3_ESM.pdf]

Counting using deep learning regression gives value to ecological surveys.

Jeroen PA Hoekendijk, Benjamin Kellenberger, Geert Aarts, Sophie Brasseur, Suzanne SH Poiesz, and Devis Tuia.

Supplementary materials S3

In this Supplementary materials S3, we compare how our seal 'Step 1 model' compares to a model trained using a random training set, with the same number of images as 'seal subset 1', but sampled across the full distribution (including images with a high number of seals, similar as 'seal subset 2').

For this new model, we randomly selected 787 images from 'seal subset 1' and 'seal subset 2' combined. The 787 images in this new subset, named 'seal subset 3', now also include 32 images with 100+ seals. This 'seal subset 3' was then used to train a regressor, analogue to the 'Step 1 model' training described in the manuscript (but covering the whole range of animal counts). The resulting 'Step 3 model' slightly improved when compared to the 'Step 1 model' (RMSE = 22.3 instead of RMSE = 23.5) but is still performing worse than the Step 2 model (RMSE = 19.0). Therefore, we argue that the increased performance can mostly be attributed to the iterative approach to model training (and an increased amount of training images) and not just to including the same types of images that appear in the test set. However, these effects are not mutually exclusive, and probably all contribute to the performance increase in the 'Step 2 model'.

	**Step 1**	**Step 3**	**Step 2**
**Number of training images**	787	787	878
**RMSE**	23.5	22.3	19.0
**R^2**	0.7	0.7	0.8

Supplementary Table S3.1. Numerical results on the test set of the seal counting application of the various models.
